# Health service resilience in Yobe state, Nigeria in the context of the Boko Haram insurgency: a systems dynamics analysis using group model building

**DOI:** 10.1186/s13031-015-0056-3

**Published:** 2015-10-05

**Authors:** Alastair K. Ager, Martina Lembani, Abdulaziz Mohammed, Garba Mohammed Ashir, Ahmad Abdulwahab, Helen de Pinho, Peter Delobelle, Christina Zarowsky

**Affiliations:** Mailman School of Public Health, Columbia University, New York, NY USA; Institute for International Health and Development, Queen Margaret University, Edinburgh, UK; School of Public Health, University of the Western Cape, Cape Town, South Africa; PRRINN-MNCH, Kano, Nigeria; University of Montreal Hospital Research Centre, School of Public Health, University of Montreal, Montreal, QC Canada

**Keywords:** Health systems, Systems dynamics, Group model building, Causal loop diagrams, Conflict, Insurgency, Insecurity, Service provision, Quality, Political will, Community cohesion, Staff commitment, Staff motivation

## Abstract

**Background:**

Yobe State has faced severe disruption of its health service as a result of the Boko Haram insurgency. A systems dynamics analysis was conducted to identify key pathways of threat to provision and emerging pathways of response and adaptation.

**Methods:**

Structured interviews were conducted with 39 stakeholders from three local government areas selected to represent the diversity of conflict experience across the state: Damaturu, Fune and Nguru, and with four officers of the PRRINN-MNCH program providing technical assistance for primary care development in the state. A group model building session was convened with 11 senior stakeholders, which used participatory scripts to review thematic analysis of interviews and develop a preliminary systems model linking identified variables.

**Results:**

Population migration and transport restrictions have substantially impacted access to health provision. The human resource for health capability of the state has been severely diminished through the outward migration of (especially non-indigenous) health workers and the suspension of programmes providing external technical assistance. The political will of the Yobe State government to strengthen health provision — through lifting a moratorium on recruitment and providing incentives for retention and support of staff — has supported a recovery of health systems functioning. Policies of free-drug provision and decentralized drug supply appear to have been protective of the operation of the health system. Community resources and cohesion have been significant assets in combatting the impacts of the insurgency on service utilization and quality. Staff commitment and motivation — particularly amongst staff indigenous to the state — has protected health care quality and enabled flexibility of human resource deployment.

**Conclusions:**

A systems analysis using participatory group model building provided a mechanism to identify key pathways of threat and adaptation with regard to health service functioning. Generalizable systems characteristics supportive of resilience are suggested, and linked to wider discussion of the role of factors such as diversity, self-regulation and integration.

## Background

### Health systems resilience in contexts of adversity

Resilience has emerged as the dominant concept underpinning development assistance and humanitarian support in nations vulnerable—through conflict or natural disaster — to crisis [1–3]. DFID has defined resilience as ‘the ability…to manage change, by maintaining or transforming…standards in the face of shocks or stresses....without compromising… long-term prospects” [4]. Fostering a complex adaptive systems approach, it is recognized that ‘the ability of the system or process to deal with the shock or stress is based on the levels of exposure, the levels of sensitivity and adaptive capacities’ [4].

UNICEF has taken a leading role in seeking to specify such adaptive systems capacities, specifying the role of flexibility; diversity; adaptive learning; collective action and cohesion; and self-reliance [5]. Oxfam has posited similar factors to be characteristic of resilient systems: diversity; connectivity; utilizing different forms of knowledge; redundancy; equality and inclusivity; and high levels of social cohesion and capital [6]. Resilience is one of the three framing concepts of the DFID strategy on humanitarian assistance emerging from response to the 2011 UK parliamentary Humanitarian Emergency Response Review [4]. More recently resilience has come to the fore as a construct relevant to understanding the basis for health services continuing in contexts of major adversity, most notably in the context of health systems in West Africa and management of the Ebola virus outbreak [7, 8].

As a construct, resilience is, however, not without critique. For example, its weak operationalization has frequently been noted [9]. Concerns have also been expressed regarding potential blindness to imbalances in power reflected in technical analysis of sources of resilience [10]. Nonetheless, there is wide recognition that resilience potentially provides a valuable framework for policy and practice on the basis of its focus on developing the capacities of populations and anticipating ‘shocks’ to systems. In particular, the capacity to ‘bounce back’ from adversity is increasingly being conceptualized as the response of complex adaptive systems to experienced shocks. Work from a wide range of disciplines — ranging from agriculture and climate science [11–13], through public health and community development [14–16] to anthropology and psychology [17, 18] — is seeing the behaviour of complex adaptive systems in adverse conditions as core to understanding resilience. That is, resilience is not so much the culmination of a number of additive ‘protective factors’ but the outcome of a range of interacting systems and influences, operating at a variety of levels. Such an insight requires the deployment of analytic tools and frameworks suited to the analysis of complex adaptive systems, such as dynamic systems modelling.

Systems dynamics has been proposed as a perspective to address the dynamic complexity which is characteristic of many public health issues [19, 20]. Although there are a wide range of approaches to systems dynamics modelling, [21] these generally share the premise that complex systems behavior results from the interplay of feedback loops, multiple interconnections, non-linearities, time delays, stocks, and flows that all occur within a system, which can be mapped and, in some circumstances, modelled using computer simulation [22]. System dynamics modelling may draw upon both qualitative and quantitative data relevant for exploring the dynamic context of health care policy interventions [23]. Although the development of simulations is an end-product of much systems modelling, the core goal of such efforts is an increased understanding of a problem and of the system in which it is taking place.[24] Group model building (GMB) has emerged as a particularly promising approach, in which models are developed to reflect diverse stakeholders’ views of the problem, often as part of an ongoing action research agenda [25, 26]. In particular, Hovmand and colleagues have shown the value of participatory group model building as a mechanism for eliciting the mental models that shape stakeholders' behaviour [27].

This study formed part of a work programme^1^ which explicitly extended the use of group model building to understand, predict and potentially influence the processes which support the resilience of health systems in contexts of adversity. While grounded in settings of particular political instability, the work was conceived to be of relevance to health systems facing diverse forms of adversity. Conducting analysis through a method involving intensive, participatory consultation with stakeholders and representation and refinement of models using graphical systems tools was seen to offer a particularly effective means of exploring the determinants of systems vulnerability and resilience. The work programme covered three case studies, focused on Cote d’Ivoire, Northern Nigeria, and Eastern Cape province in South Africa. This paper reports on the Northern Nigeria study.

This study was completed in collaboration with the Partnership for Reviving Routine Immunization in Northern Nigeria and Maternal Newborn Child Health (PRRINN-MNCH) programme. PRRINN-MNCH had been operating in Northern Nigeria since 2008 with support from the UK Department of International Development (DFID) and the State Department of the Norwegian Government. The programme aimed to impact maternal, newborn and child health in Northern Nigeria by improving access to maternal and child health, including routine immunization services, in four States: Jigawa, Katsina, Zamfara and Yobe. Of these four states it is the latter that has been most significantly impacted by the Boko Haram insurgency. It was on this basis that Yobe was selected as the geographical focus for the case study.

### The Boko Haram insurgency in Yobe State

Yobe State^2^ has experienced attacks from the militant group popularly known as Boko Haram (‘against Western education’) since 2011. Initially the group targeted police and churches, but from 2012 the attacks — bombings, military raids and robberies — expanded to target mosques, schools, hospitals, and banks.

The neighbouring states of Borno and Adamawa were similarly affected and a state of emergency was declared in these states in May 2013. The situation created much fear and panic among citizens. People left their homes and migrated to what were seen as safer locations. This significantly disrupted livelihoods as people were displaced from their land and other places of work. Security concerns and travel restrictions imposed by the state severely curtailed opportunities for commerce. As the insurgency evolved, Boko Haram started burning villages and kidnapping community leaders. At the time of writing, Yobe state is still in a declared state of emergency. Many organizations have terminated their operations in the state, while those who continued working (such as PRRINN-MNCH) have done so with key activities (such as consultation meetings and trainings) conducted outside the state. This has increased the costs of programme implementation significantly.

The health system has been majorly affected by these events. Health workers, especially those not indigenous to Yobe, have fled to other states. Indigenous workers are generally reported to have only temporarily abandoned their work places during spikes of insecurity, returning when the situation improved. Some health facilities have been directly attacked, with insurgents taking away drugs, hospital equipment, ambulances and other vehicles.

In some instances health workers have been abducted, while others have been killed by blasts when traveling to or from work. Some hospitals have had to be closed for some time until the local situation normalized. Movement for health workers and patients to access health facilities has been a major challenge due to curfews imposed by the security services (the Joint Task Force, JTF). Curfews have been regularly declared and significantly restricted movement. When curfews are lifted, delays are frequent because of multiple checkpoints mounted on the roads. On initial deployment of the JTF, with no established relationship between locals and the largely non-indigenous security forces, harassment was reportedly common, though cooperation grew with greater mutual familiarity.

Other aspects of counter-insurgency response — such as the banning of motorcycles (a favored means of transport for Boko Haram raids), the prohibition of the growing of any plant over 1 m high (seen to provide cover for insurgents) and the interruption of cellphone service (to restrict contact between insurgents during major anti-insurgency operations) — have also contributed to a challenging environment for the functioning of, and access to, health services.

## Methods

The study was structured according to four distinct phases of activity, following the conventions of systems dynamics analysis adopting a group model building approach [27]. The first phase was focused on the development of a scenario definition (defining the boundaries of the case study) and subsequent interviews with key informants. The scenario definition was constructed on the basis of preliminary planning discussions with key PRRINN-MNCH staff, including the National Programme Manager, the Deputy National Programme Manager and the Yobe State Team Manager.

This confirmed Yobe as the geographical focus of the study and primary health care provision as the principal service focus of analysis. This also informed a structured interview guide regarding major challenges faced in the course of the insurgency and strategies to address them.

Given logistical constraints on interview coverage and the varied impact of the insurgency in different parts of the state, the scenario definition further focused detailed analysis on three of Yobe’s 17 Local Government Areas (LGAs). These comprised: Damaturu (the location of the state capital, which has been both directly — through attacks — and indirectly — through migration — impacted by the insurgency), Fune (a rural area directly impacted by the insurgency at varying times) and Nguru (an area that has experienced no direct attacks, but has been indirectly impacted through significant inward migration).

Thirty-nine interviews were conducted across these three LGAs in May through June 2014. Interviewees included health managers, local government officials, health facility personnel and patients. In addition, four PRRINN-MNCH staff were formally interviewed: the National Programme Manager, the Deputy National Programme Manager, the Yobe State Team Manager and the Operations Research Coordinator. Thus a total of 43 interview transcripts were available to inform the second phase of the study.

This second phase involved preliminary data analysis and planning. Two members of the research team and two PRRINN-MNCH staff based in Yobe served as the core planning team. Each member of the planning team reviewed all transcripts of interviews and then independently identified key themes emerging from them. The members of the planning team then worked together to group themes and consolidate them into defined variables. The planning team then developed an interrelationship diagraph (IRD) identifying what was suggested, from the data reviewed, to be major causal linkages between these variables, as well as the key drivers and outcomes within the system. Informed by the IRD, the planning group then specified a ‘seed model’ as a starting point for model building at the group model building (GMB) workshop. Finally the team developed a series of scripts to structure discussion at that workshop. The scripts are a detailed description of the processes followed during GMB sessions (based upon the work of Hovmand and others [24–27]).

The third phase involved the GMB workshop itself, held in June 2014. This was a one-day session for 11 participants, identified by the planning team as representing key perspectives on the functioning of the health system in Yobe during the insurgency. This included representatives of the State Ministry of Health, the State Primary Health Care Management Board, and Health Training Institutions (HTIs); the Medical Director of Damaturu Specialist Hospital; a local engagement officer who had conducted key informant interviews; and four senior staff of PRRINN-MNCH. Scripts were used to: elicit a shared understanding of key characteristics of the insurgency (using a ‘rich picture’ exercise in which participants created a shared drawing of the situation under consideration); review, confirm, illustrate and elaborate upon the listing of variables identified by the planning team; link these variables, building upon the provided ‘seed model’, by specifying two preliminary systems models accounting for experience in a specified urban and rural location in the state respectively; and consolidate these two models into a single causal loop diagram with the aid of Vensim graphical software^3^. The GMB session closed with a reflection on the insights prompted by the preceding analysis and the availability of data to facilitate quantitative refinement of the model.

Phase four of the study involved processes of documentation, refinement and dissemination. A full transcript of GMB discussions was produced, and the data used to elaborate the analysis which follows. Health Management Information System (HMIS) data for particular locations — Damaturu LGA, Gujba LGA (a proxy for Fune LGA, with similar insurgency experience) and Nguru LGA — was extracted and consolidated. Along with data from other sources (such as the Yobe State Human Resources Information System) this was principally used for triangulation of statements made during interviews and the GMB. Data was not available with sufficient completeness and reliability to document changes in key variables over time and complete sensitivity analyses. Refinement therefore focused on clarification of key pathways of threat and response, and identification of key leverage points. This analysis was reviewed and validated through a dissemination workshop held with local stakeholders in Damaturu in December 2014.

## Results

**Fig. 1:**
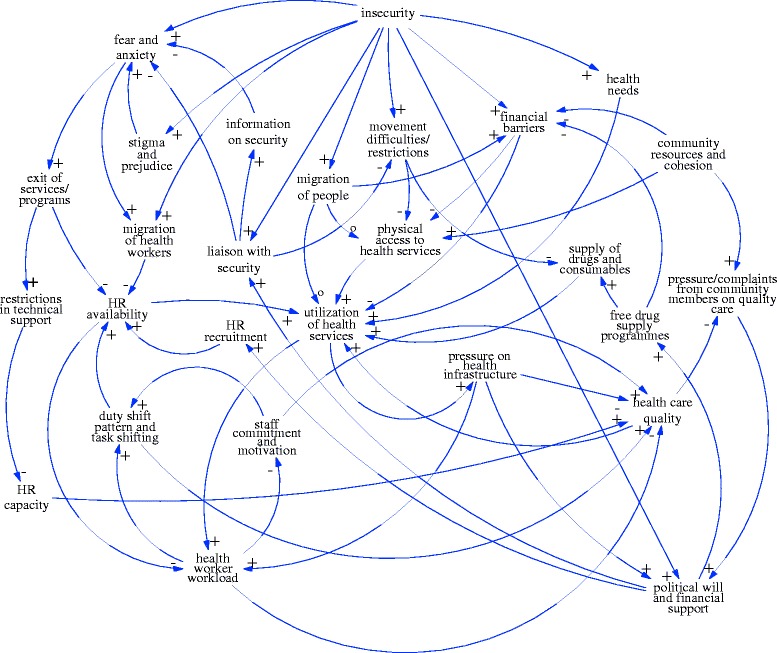
Causal loop diagram of factors influencing health utilization and service quality during the insurgency in Yobe developed through group model building

Fifteen variables were confirmed by GMB participants as key in accounting for health systems functioning during the crisis. These are considered, in turn, below. During the course of modeling, an additional 12 variables were specified as intervening factors clarifying linkages between variables, with staff commitment and motivation, the most elaborated. The linkages identified between all these variables are shown in Fig. 1, the causal loop diagram developed by participants during the GMB exercise.

### Insecurity

Between June 2011 and August 2014 there were numerous documented attacks by Boko Haram within Yobe state resulting in an estimated 1341 fatalities [28]. Incidents peaked in October 2012, with over 12 reported attacks and 140 fatalities. There were 252 deaths in Yobe state in the first 6 months of 2014 alone [29]. Interviews documented many incidents vividly illustrating this prevailing insecurity: One health facility worker, for example, reported:

When Dogon Kuka was attacked, people ran away and the health facility was closed for almost three months. One security guard was killed in his house.

Another health worker told of how one day gun shots started when she was in the office and there was no way she could move to her house easily. All the staff lay on the ground, weeping on the bare floor tiles.

Due to such insecurity, many people felt compelled to migrate out of their villages, and health workers left their workstations for safer places. Insecurity also created movement challenges, as people were afraid of being attacked when traveling because insurgents often blocked the roads and attacked people.

During the GMB session one participant reported an incident regarding some health workers who were attacked after the prevailing 6.00 pm curfew because they had been called to render services at the hospital due to an emergency. Even though they had IDs to allow them to travel during curfew hours, the Nigerian police had physically harassed them. There were a number of reports of the police abusing their power through assaults. This situation was aggravated when the ‘Escobar’^7^ phenomenon was at its peak, since one could not differentiate between the official security forces and criminal gangs because they were all wearing uniforms, carrying guns, and driving HILUX vehicles. Such incidents were reported to have been at their peak during 2012 and 2013.

### Migration of people

As the bombings, abductions and killings became wider and more indiscriminate in their scope, increasing numbers of people relocated to what were perceived to be safer areas. When attacks were concentrated on urban settings, migration was to rural areas. When urban centers such as Damaturu became more secure, attacks shifted to more rural areas, and migration was towards the towns. For example, one of the interviewees in Damaturu reported:

That time around, the patients have even migrated and left the town because it is our catchment area, Damaturu, that was affected by the activities of the insurgents most especially in the Gwange area.

A GMB participant expressed similar sentiments and stated that in the beginning the attacks were concentrated in Damaturu and people were moving from Damaturu to various places. However the situation changed when Damaturu was heavily secured by armed forces, the insurgents turned to the rural areas, and the migration patterns reversed.

### Migration of health workers

Health workers were not spared in the killings and threats. Indigenous workers generally abandoned their workplace temporarily and returned whenever the situation calmed down. However, most non-indigenous workers left permanently. Records gathered from the Yobe state Human Resource Information System (HRIS) indicate that the most affected cadres of staff were doctors and nurses/midwives as compared to other staff as presented in Tables 1 and 2.

**Table 1:** Yobe state health workers by selected local government area (LGA)

LGA	Year	
	2010	2014
Gujba	529	292
Damaturu	468	583
Potiskum	368	450

Number of health workers (other than doctors and nurses/midwives) in 2010 and 2014 for specified LGAs. Source: Yobe State Human Resources Information System

**Table 2:** Yobe state health workers by hospital

Hospital (LGA)	Doctors		Nurses/Midwives	
	2010	2014	2010	2014
General Hospital Buni Yadi (Gujba)	3	0	33	25
Sani Abatcha State Specialist Hospital (Damaturu)	24	17	107	27
General Hospital Potiskum (Potiskum)	11	1	86	26

Number of doctors and nurses/midwives in post in 2010 and 2014 for specified hospitals in Yobe state. Source: Yobe State Human Resources Information System

With the exception of Gujba LGA, Table 1 indicates that the number of health workers in other cadres had increased in 2014 compared to 2010, which is an indication of a higher retention during the crisis.

However, figures in Table 2 indicate a significant decline in the number of doctors and professional nurses/midwives across each of the three LGAs over the same period. For example, the general hospital in Buni Yadi lost all its three doctors it had in 2010 and there was no doctor left at the hospital by June 2014. The general hospital at Potiskum lost about 90 % of its doctors over the same period. Only Sani Abatcha State Specialist hospital seemed to have significantly fared better, losing only 30 % of its physicians. 2014 figures are likely to reflect an upward trend from 2013 when the State Governor lifted an embargo on recruitment in recognition of the acute shortages that had arisen (HRIS data is not available for intervening years). GMB participants stated that more doctors are arriving and picking up jobs in hospitals now, hence the increase.

The trend for professional nurses/midwives is similar. For instance, Sani Abatcha Specialist Hospital lost 75 % of its professional nurses/midwives while the general hospital in Potiskum lost about 70 % of this cadre. Only General Hospital Buni Yadi fared better, losing only about 25 % of such staff. Midwives were especially affected because most of them were from other states deployed through the Midwives Service Scheme (MSS) and almost all of them left the state at some point.

Key informants confirmed that during the crisis, most health workers did not want to stay in Yobe state. One participant at the GMB session  stated:

In the hostel, a lot of medical doctors left, the National Youth Service Corps (NYSCs) do not come to Yobe at all, not even Damaturu. A lot of non-indigenes left, a lot of indigenes who are outside Yobe couldn’t come. But now it has been reversed. There were only four NYSCs who reported initially but now there are 16.

Participants reported that more NYSCs were currently coming to take up posts in Yobe state.

### Movement challenges/restrictions

The many dangerous roads where insurgents could mount ambushes presented significant challenges regarding movement within the state. However, counter-insurgency measures also led to major travel restrictions. Curfews were sometimes set to begin as early as 4 pm, creating major logistical travel difficulties. At times the JTF blocked roads that were considered too dangerous for people to travel on. Diversions, significantly adding to journey times, would generally be provided, but on occasions no alternative routes were available, preventing all travel. Although some categories of health staff were provided with IDs to ease movements, there were still some challenges. One participant noted:

The ‘Road Pass’ helps most times but still sometimes it wouldn’t because it seems one is exposing himself and you don’t know who else is watching, even the security. It may come across like a particular person thinks he is special and they may follow you and attack you. So at times you may have the pass and may not want to use it.

Another participant added to this experience stating:

Sometimes you have to bring your stethoscope out of your car. Some of us have a ‘Road Pass’ to enable us pass the queue but it does not prevent a search. When there is high tension, when you go and meet the security officer, they don’t understand but some of them that understand may agree to search you and let you go.

### Supplies of drugs and consumables

GMB participants confirmed the view emerging from stakeholder interviews that availability of drugs and other consumables at facilities was, in general, not severely disrupted by the insurgency. The State Ministry of Health and facility managers minimized stock outs by changing deliveries from the usual 1-month supply to a 3-month supply.

Additionally, facilities were encouraged to order new stocks when they had 50 % of the drug stocks remaining. In the context of mass casualty events, some short-term drug stock-outs were reported, but restocking was secured swiftly. The decentralized nature of the drug supply chain, with drugs managed at the state rather than federal level, made it much easier to transport drugs to and from the Damaturu drug store whenever transport routes were secure. Flexibility in local supply routes helped secure supplies when circumstances were volatile.

The GMB participants and key informant interviewees both reported drugs being brought somewhere close to a facility for collection by facility personnel once access was possible. There was some evidence of commercial drug supplies being more severely impacted due to transport difficulties and loss of consumers' purchasing power. One local drug vendor reported that to get a lorry to take their drugs to Damaturu sometimes took up to 2 weeks. Even when they got the transport, the price was double because the drivers didn’t want to go to Damaturu due to the insecurity. Another supplier reported:

I used to sell an average of 30,000 Naira per day but with the insurgency, I could only sell about 10,000 Naira a whole week.

### Physical access to health services

Physical accessibility of health facilities was one of the most important factors influencing patient utilization of health services. Interviewed patients stated that during the critical periods of the insurgency, it was extremely difficult to get to a health facility due to travel restrictions and fear of being attacked. One patient noted:

As you know, we can’t come to hospital easily because of their activities…people have suffered a lot because of the insecurity, even if you are not well and you want to come to the hospital it is a big problem…if you intend to go to the hospital you are thinking of what is going to happen to you on the way.

Some pregnant women were reported to have died as a result of complications during delivery due to failure or delay in accessing health facility care. One of the participants in the GMB session testified about his close friend whose wife died in labour at home because she could not be taken to the hospital due to travel challenges.

At some point, arrangements were made with the JTF for security personnel to collect patients from their homes and escort them to the health facilities when contacted through a medical emergency telephone number. Unfortunately, this service had later to be discontinued after insurgents feigned illness and then ambushed the military forces coming to attend them. This had resulted in an arrangement where those telephoning the emergency number and providing a description of their vehicle were allowed to use their own cars to access a health facility during curfew hours. The JTF then monitored the passage of the vehicle from a position of cover.

### Financial barriers

Movement restrictions have limited people’s access to gainful activities, hampering their financial capacities. People could not trade their commodities for fear of attacks. From the early months of the insurgency, farming of certain crops such as maize was banned so that insurgents could not use those fields as cover to launch their attacks. Also, due to curfews and direct attacks, banks were only open for a short period of time and some were completely closed. There were also restrictions on interbank transfers. Even those who had money in their bank accounts could not easily access this money to transact business.

A number of interviewees noted that the financial barriers to service access during the insurgency were, however, significantly less than they would have been without the introduction in 2009 of a state drug subsidy scheme. This ensured that all pregnant women and children under-five had access to free drugs. All drugs and materials required to treat a medical emergency, such as a shooting or bombing, were also covered. These arrangements, and the separate ‘free MNCH’ programme operating in some facilities, thus moderated the health impact of the disruption of livelihoods resulting from the insurgency.

### Utilization of services

Interviews suggested that the level of security influenced the use of services in a complex manner. If the number of attacks was high in a particular area, the utilization of services was low because patients were afraid to travel to the health facilities. A medical doctor from one of the most affected areas stated that most patients that came to seek care did so when they were critically ill and required urgent medical attention. Patients who were not severely sick would defer or decline attendance due to insecurity. Mothers who had appointments for routine immunization, for example, often missed their appointments.

However, if an attack involved large numbers of casualties then health facilities nearby would be filled with patients injured during an attack. Areas that experienced fewer or no attacks reported a general increase in patient attendance because people had migrated to those places.

**Fig. 2:**
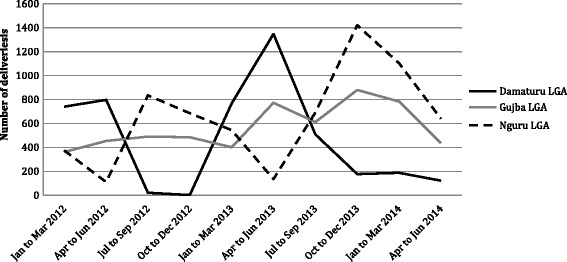
Trends in facility deliveries: total facility deliveries per quarter for specified LGAs. Source: Yobe State HMIS

**Fig. 3:**
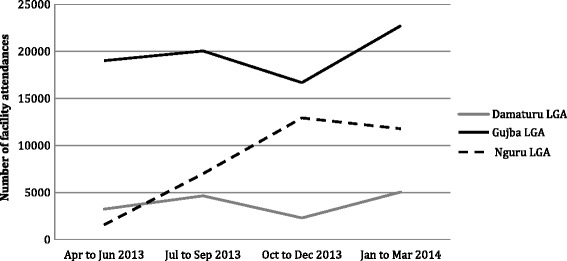
Trends in facility attendance: total facility attendance per quarter for specified LGAs. Source: Yobe State HMIS

HMIS data on facility attendance confirmed great fluctuations due to insurgency activities and related counter-insurgency measures. For example, Damaturu has at times been a major focus of attacks, but through counter-insurgency measures has at other times been seen as a relatively safe haven. Figs. 2 and 3 illustrate this pattern of varying patient attendance for the three LGAs for which HMIS data was reviewed.

Figure 2 presents trends in total number of facility deliveries per quarter in three LGAs from January 2012 to June 2014. The pattern indicates that whenever there was a decrease in facility deliveries in Damaturu, there was a corresponding increase of facility deliveries in Nguru. For example, during the quarter July to September 2012, the number of facility attended deliveries in Damaturu declined to 21 deliveries from 797 in the previous quarter, while in Nguru the numbers increased to 835 deliveries against 111 deliveries over the same period. By April to June 2013 Damaturu had experienced a sharp increase in facility deliveries while in Nguru the numbers reduced to previous levels. By late 2013 the pattern had reversed again, with deliveries in Damaturu steeply declining and those in Nguru rising again. Attended deliveries in Gujba were relatively stable, with some fluctuation, during this period.

Similarly, Fig. 3 shows total recorded facility attendance across LGAs from April 2013 to June 2014. Reporting from facilities was not complete during this period, so reported rates are assumed to be an underestimate of total attendance. However, with comparable reporting rates across the three LGAs comparison of trends across the different settings is likely valid.

This data suggests a marked increase in service utilization in Nguru during the course of 2013 (in line with the increase in attended deliveries during this period). With no reported insurgent attacks in Nguru, this appears to reflect internal migration within the state to an area perceived as safe during a period of major insecurity elsewhere.

### Health worker workload

As most non-indigenous health workers left Yobe state, fewer health workers were left to attend to patients. For example, as noted earlier, the number of nurses/midwives at the specialist hospital in Damaturu dropped from 107 in 2010 before the insurgency to 27 during the insurgency period in early 2014.

Increased patient numbers at times of inward migration or mass casualty incidents exacerbated the impact of this on workload. For instance, through November and December 2011, Damaturu experienced a number of intense attacks where over 100 people were killed, each resulting in many casualties requiring urgent treatment. With increased workload the quality of services was compromised because the few available staff could not effectively attend to all the patients. Interviews documented reports of patient deaths in the hospital attributed to delays in treatment due to inadequate staffing levels. One nurse noted:

I am a nurse by profession, so I have restrictions, I am not supposed to be clerking patients, but when they meet me, I have to step out to do the function of a medical officer and also nursing work…the pressure on me will definitely undermine efficiency.

### Pressure on health infrastructure

Increased utilization of services at facilities also created pressure on health infrastructure. Primary health care workers regularly signaled the potential impact of this on service quality:

the quality of services is not at all what I could call standard because we have a lot of crowdedness due to the turnout of the patients.

These pressures affected secondary and tertiary care also. Bed space was frequently inadequate when patient attendance increased beyond planned health facility capacity. Mortuary capacity was reported to have often been inadequate to meet demand following mass casualty incidents. Hospitals had to ask the environmental department to assist in clearing away the dead bodies. Pressure on health infrastructure was so severe that it prompted the state governor to intervene to release funds to construct more mortuaries and extend hospital wards.

### Liaison with security services

Liaison with security forces was key during the crisis to enable essential movement of health workers during curfew hours. Doctors were sometimes called to go to the hospital and help with emergencies but due to curfews they would be restricted in their movement. The Ministry of Health made arrangements with the JTF to allow health workers to go to work during curfew hours through prior arrangement with local security forces.

This was subsequently facilitated by the provision of identity cards to show to the security personnel when crossing checkpoints. However, health workers reported sometimes being reluctant to show IDs for fear that this exposed their identity to potential insurgents who on occasions targeted killings of government personnel.

Community members also liaised with security forces whenever they needed to transport a sick person to the hospital or, more generally, if they wanted to report information about insurgents in their community. However, those seen talking to security forces were considered a direct target of insurgents, with one participant in the GMB session claiming:

If they see you with the JTF number in your cellphone, or you are talking to a security officer, you are dead.

State officials would also regularly liaise with security forces to seek to reduce curfew hours to enable citizens to move around more freely and go about their business.

### Duty shift patterns and task shifting

The restrictions in movement due to curfews created challenges in the duty shift of health workers who were scheduled to travel to work at restricted times. To resolve this challenge, the Hospitals Management Board convened senior physicians and nurses and made a formal change to the health worker duty shifts. Instead of three 8-hour shifts, two 12-hour shifts were established. This ensured continuity of staffing at facilities in a manner achievable with curfew restrictions.

Other less formal adjustments to working practices were also reported. In the context of the crisis, health workers were reported to have assumed duties for which they were not fully trained and licensed: a form of informal task-shifting. Such flexibility in role allocations was widely perceived as a necessity in the context of the prevailing crisis, and a move that has demonstrated the significant capability of less senior cadres.

### Financial support

As a result of the crisis the health sector required additional financial support to address the increased demand for health services because of casualties. As noted earlier, the State government was already funding free drug programmes for specific groups before the insurgency, and this arrangement extended to covering the costs of those attending for emergency intervention following insurgent attacks.

Given State responsibilities for funding contributions to the JTF security operation, Yobe state budgets for all sectors, including health, were put under severe pressure. Longer term development projects (e.g. health facility construction) were postponed, but generally finance for the health sector was robustly supported. In 2013 the health sector absorbed 97.3 % of its allocated budget, which represented a budgetary allocation double of that allocated for the health sector in 2011 when the crisis had just begun (and of which only 60 % had ultimately been disbursed).

### Political will

Stakeholders attributed financial support to the ‘political will’ exhibited by the State Governor. His visits to hospitals and other facilities in the aftermath of major attacks were clearly appreciated. One participant at the GBM session noted

Actually, the renovation to some extent was triggered by the insurgency. The Governor was not even happy about the capacity of the infrastructure and that is why he declared a State of emergency on the health sector. I recall the Governor having been around more than ten times, which is unprecedented. He comes at will, without being announced, just to see everything that he needs to see and for some years back sometimes it was hard to see the Governor.

Additionally the state provided security personnel in the cities and also facilitated removal of some of the roadblocks to support movement. More specifically to the health sector, on appreciating the loss of capacity in the health workforce with the loss of non-indigenous staff, the state sanctioned the lifting of an established embargo on employment to the health sector. Further, the state provided incentives to health workers such as offering furnished houses and ensuring regular and on-time salary payment, which was generally secured despite all the challenges with the banking system.

### Community resources/cohesion

Community support was regarded as a key factor that helped community members to cope during the crisis. This involved providing spiritual, emotional, and social support to each other. Most of the interviewees mentioned that they survived by faith and through prayers, indicating that religion had played a major role in holding the people together, giving them hope to go on and endure the crisis and its suffering. Community members organized transport for those who needed to access health facility services, and provided shelter to those who had abandoned their homes due to insecurity. One GMB participant recounted how he had moved his family to a safer area but, in the context of general fear and uncertainty, had recognized the need to build the trust of the local community by attending Friday prayers in that area.

Community members were a key source of information regarding the insurgents. They would alert the health workers not to conduct activities, especially immunization campaigns, whenever the insurgents were operating within, or close by, their local area.

Co-ordination of immunization is done through community leaders because they are the ones that know when there is threat of insurgency around them since these health workers are with them, they work hand in hand with them.

The community leaders are usually the one’s in the center of co-ordination. They are the ones who usually see [the insurgents] when they are passing. The health clinic has nothing for now as a way of coordination.

We have tried to work hand in hand with all arms…we had a series of meetings with emphasis on community engagement in all the health interventions. When you connect very well with the community and traditional leaders, you will get adequate information including security information on how best they can deliver services. Sometimes if health facilities are closed health workers are in their houses, they can render services within their community to the level that is feasible…..

### Staff commitment and motivation

The preceding quotation highlights the importance of one variable, not initially identified during thematic analysis, which participants at the GMB considered important for explanatory purposes: staff commitment and motivation. Interviews and discussion indicated that commitment was generally high among the health workers remaining in post through the insurgency. Health workers reported encountering many challenges but this was usually reported to have not deterred them from providing services to the patients, although they often worked under fear. Most of the facility in-charge persons reported that they used to encourage and motivate their staff to ensure their availability at the facility and this helped the health facilities to cope during the crisis period. One facility in-charge reported:

Since I am in-charge of the facility, I don’t think there was any time I did not come to work and I always organized my staff to come to work.

Patients who were interviewed backed up these assertions. They also reported on the level of commitment with which the health workers operated as stated by one patient:

Thank God the truth is that the health workers are trying their best to provide quality services.

Another patient reported:

The quality of service is good always and we find the health workers there…before the insurgency the number of health workers were more than now but they are trying, even now.

There were reports of health worker commitment in the face of significant danger. One immunization officer talked of when a team had been intercepted by Boko Haram:

Insurgents came and warned them to stop the work because it is evil. If not, they will kill them next time they see them doing it. The women thanked them but continued with their work after the[y]… had left. One method they adopted is hiding vaccine carriers in polythene bags… putting the vaccine in between luggage so as to hide it from anyone watching them.

## Discussion

**Fig. 4:**
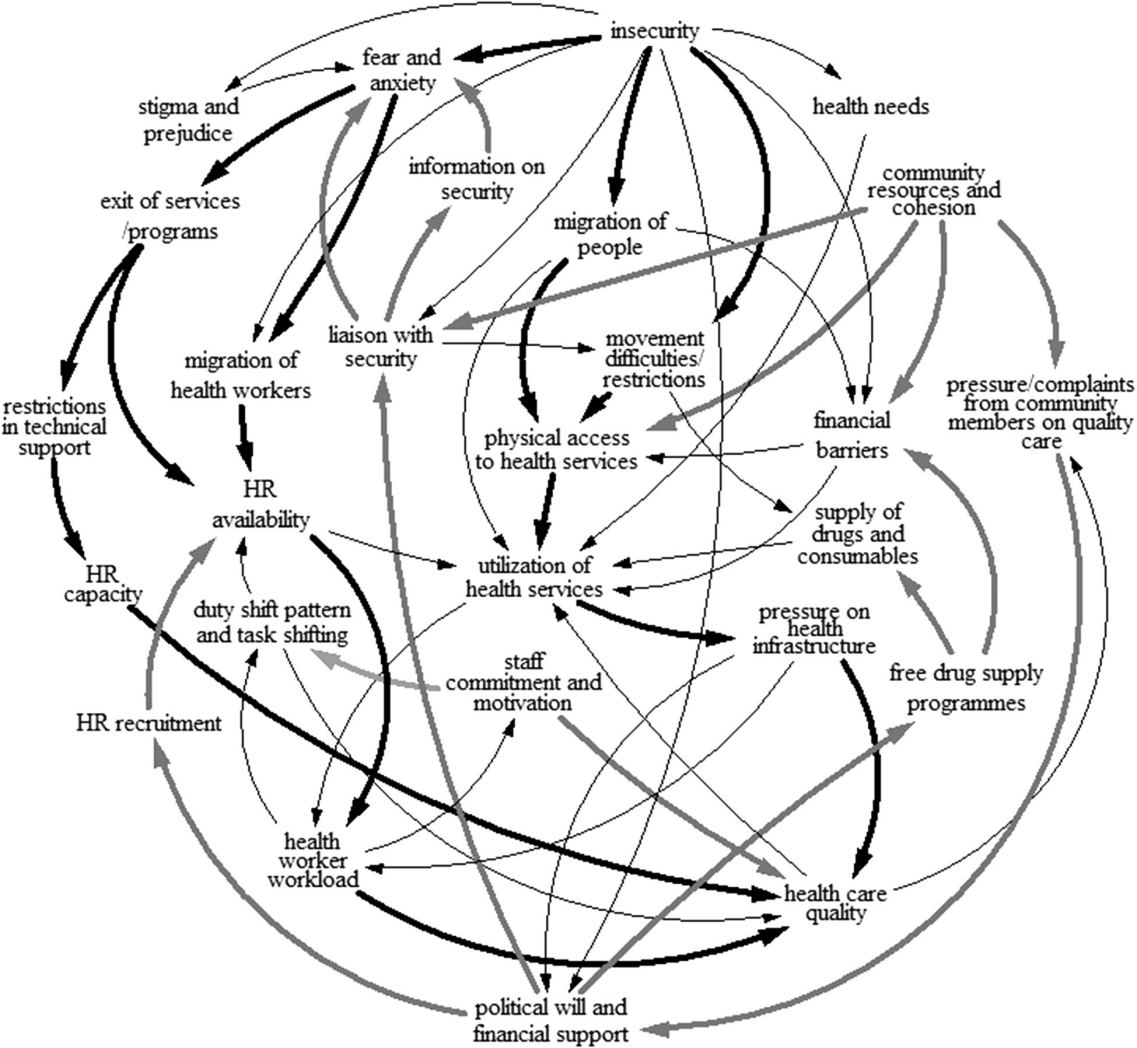
Systems model developed by stakeholders in Yobe and refined to show key pathways of threat and response (key pathways of threat in black; pathways of adaptation in grey)

As noted earlier, the Yobe State HMIS did not provide data of sufficient completeness and reliability to allow construction of a simulation model based upon the linkages suggested by Fig. 1 and thus exploration of sensitivity analysis with respect to key variables. However, review of variables and linkages in the context of the recorded GMB discussion, further review of interview transcripts, and triangulation using HMIS data together supported the identification of major explanatory pathways amongst the multiple linkages indicated by the initial causal loop analysis. These pathways may be understood as feedback loops extending across multiple domains that have influence on variables of particular concern for our analysis: utilization of health services and health care quality. Rather than focusing on specific balancing or reinforcing loops, points of influence and leverage were — through stakeholder consultation – found to be most effectively identified by specifying key pathways of threat and response. Fig. 4 shows the explanatory structure that evolved, with key pathways of threat and disruption (marked in black) and of response and adaptation (marked in grey) indicated.

The threat pathway to the left of the figure shows the impact of insecurity on service utilization and health care quality through depletion of the human resources available to the health system. Mediated by fear and anxiety, this pathway reflects both the direct loss of personnel to the health system and the loss of capacity due to the withdrawal of technical assistance to support health workers. The threat pathway in the central section of the figure indicates the influence of insecurity on health service utilization and health care quality through restricting access to health services, either directly through transport difficulties or indirectly as a result of population displacement.

The systems analysis facilitated by GMB thus indicated two major pathways of threat, both emanating from the central driver of insecurity: one fundamentally eroding human resource capacity available to the health system and one fundamentally disrupting patient access to services. Systems analysis also provided insight into the major pathways of response and adaptation to these threats, and the principal drivers of these pathways.

Three principal drivers, each responsible for a discrete pathway of systems response (marked in grey in Fig. 4), were identified. GMB participants judged what they termed political will [30, 31] on the part of State Government and other parties to have reinforced a number of pathways ameliorating systems impacts of insecurity. These included direct attempts to normalize conditions through liaison with the JTF, such as efforts to minimize transport disruption and reduce fears and anxieties. Most specifically, there were State actions directly targeting the bolstering of human resources capability, including the lifting of the recruitment moratorium and ensuring prompt remuneration for health workers. There were also pre-existing commitments to free-drug supply that was widely seen as pre-emptive of greater threats to health service provision through the insurgency (a true basis of systems resilience).

Community resources and cohesion were identified as another key driver of pathways of influence mitigating the impacts of the crisis. The community was drawn together over time to liaise with the security services, which reduced fear and anxiety and their negative impacts. Communities pooled resources (knowledge, transport and finance) to enable physical and financial access to health facilities for those in need. Communities were also seen to have played a key role in mobilizing political will, though concerted advocacy in relation to the fragility of health provision in the wake of the insurgency.

The other key pathways promoting systems resilience in the context of the ongoing crisis were seen to stem from staff commitment and motivation. Although this is difficult to objectively quantify, interviews and discussion repeatedly noted the strong commitment of indigenous health workers and its protective influence. This operated through an acceptance of a challenging shift pattern that accommodated to the prevailing curfew and taking on additional responsibilities through informal task-shifting, as well as more direct influence on quality of care.

Two other areas of Fig. 4 warrant discussion. The flow of information from and to security services was a core dynamic underpinning health systems response to the insurgency. This flow was considered to be very restricted on initial deployment of the JTF, when a lack of community awareness amongst a security force largely drawn from out-of-state created significant tensions. However, as awareness increased, so did trust and information flow, which was crucial to the passage of health personnel, patients and commodities. Coordination between the State Ministry of Health and security forces enabled health workers to be cleared faster at check-points after identifying themselves with special IDs. The JTF also provided security escorts to patients in emergencies, before insurgents hijacked the arrangement. In addition to such formal mechanisms, community health workers reported concerted coordination with community members who warned health workers when it was not safe for them to visit specific communities, for example, for immunization activities.

Finally, the supply of drugs and consumables and financial barriers and their linkage to travel and utilization are not emphasized as major pathways in Fig. 4 as interviews and GMB discussion confirmed that these had not been significant variables during the course of the insurgency. However, as noted earlier in this discussion, this may be seen as evidence of true resilience in the context of the Yobe health system: the prevailing decentralized ’push’ drug supply approach, supported by good state storage capacity in Damaturu, and the free drug supply policies introduced before the crisis both served to protect key functional capacities of the system.

The presentation and discussion of these findings with local stakeholders in Damaturu in December 2014 prompted impassioned debate. Since the time of interviews and group model building earlier in the year, insurgent activities had escalated in many parts of the state. This had led to the forced closure of a general hospital and two comprehensive health centers in LGAs exposed to recurrent Boko Haram raids. While there had been no reported destruction of facilities and health worker casualties in these areas, newly-furnished doctors’ quarters in Damaturu had been attacked and two doctors killed at close range. This had severely disrupted health service delivery in the state capital for over one month.

Discussions confirmed the relevance of the drivers of political will, community engagement and staff commitment as a basis for recovery from such assaults on the health system. The state government had recognized the importance of secure accommodation and was currently building quarters for health workers within the grounds of the hospital. The establishment of a life insurance scheme was seen to have encouraged and motivated health workers to remain in their posts, and had even attracted new graduates to seek appointments in the state. Longer term, noting the high commitment and retention associated with indigenous staff, greater access to health professional careers through enhanced training institution capacity was considered crucial.

## Conclusions

Although quantitative data was not available to pursue simulation and sensitivity analyses, systems dynamics analysis using participatory group model building provided a mechanism to identify key pathways of threat and response regarding health service functioning. Although these pathways are to some degree unique to the specific circumstances of Yobe state within the context of the ongoing insurgency, certain broader systems principles supportive of resilience are suggested. Reflecting analyses from parallel studies in Cote d’Ivoire [32] and Eastern Cape province [33], these include the value of collateral pathways, storage capacity, narrowing focus, and alignment of motivational interests. Collateral pathways refer to the availability of alternative routes to achieve a desired goal. In the context of Yobe this was exemplified by, for example, the accommodation of shift patterns to curfew requirements and the informal task-shifting that enabled key roles to be covered by available staff. Storage capacity within the system was exemplified by such processes as the increased drug stocks supplied during periods of security and the number of Yobe-born health workers available to take up posts in the state when non-indigenous staff left their positions. Narrowing focus was exemplified by the emerging emphasis on certain critical services and the occasions when health workers were withdrawn from some settings and redeployed elsewhere on the basis of prevailing levels of insecurity. Finally, aligned motivational interests were reflected in the number of reports of reduced conflict within health teams faced by a common threat and the accounts of increased community cohesion and support through the course of the insurgency.

These empirically-derived principles bear some coherence with the characteristics of resilient health systems proposed by Kruk and colleagues in their recent conceptual review in response to the Ebola epidemic in West Africa [8]. They propose awareness, diversity, self-regulation, integration and adaptation as key elements supporting health systems resilience.

Diversity, for example, references the value of multiple mechanisms to address health needs, closely corresponding to the above notion of collateral pathways. Self-regulation refers to the capability to direct the focus of services and mobilize resources, akin to notions of narrowing focus and storage capacity suggested here. Integration — bringing together diverse actors — has close parallels with the idea of aligned motivational interests. Perhaps most crucially, this analysis illustrates mechanisms of systems adaptation: ‘the ability to transform in ways that improve function in the face of highly adverse conditions’ [8]. Future work should seek to determine whether the systems features identified here are indeed a recurrent and useful framing of characteristics supportive of resilient health provision. Participatory group model building offers a potential insightful means of such explorations.

## Abbreviations

DFID: Department of International Development
GMB: Group model building
HMIS: Health Management Information System
HRIS: Human Resource Information System
HTI: Health Training Institution
IRD: Interrelationship diagraph
JTF: Joint Task Force
LGA: Local Government Area
MSS: Midwives Service Scheme
NYSCs: National Youth Service Corps
PRRINN-MNCH: Partnership for Reviving Routine Immunization in Northern Nigeria and Maternal Newborn Child Health Programme

## Footnotes

1 Health Systems Resilience: A Systems Analysis is an associate programme of the ReBUILD Consortium (www.rebuildconsortium.com).

2 See http://nigeriazipcodes.com/wp-content/uploads/2012/07/Yobe-state-Postcode-Map.jpg

3 Vensim is a software package used for developing, analysing, and packaging dynamic feedback models. See vensim.com.
